# Development and Validation of a Scoring System to Predict 2-Year Clinical Remission in Ulcerative Colitis Patients on Vedolizumab

**DOI:** 10.1093/crocol/otae068

**Published:** 2024-12-28

**Authors:** Thanaboon Chaemsupaphan, Aviv Pudipeddi, Huiyu Lin, Sudarshan Paramsothy, Viraj Kariyawasam, Melissa Kermeen, Rupert W Leong

**Affiliations:** Department of Gastroenterology and Liver Services, Concord Repatriation General Hospital, Sydney, New South Wales, Australia; Division of Gastroenterology, Department of Medicine, Siriraj Hospital, Mahidol University, Bangkok, Thailand; Department of Gastroenterology and Liver Services, Concord Repatriation General Hospital, Sydney, New South Wales, Australia; Faculty of Medicine and Health, University of Sydney, Sydney, New South Wales, Australia; Department of Gastroenterology and Liver Services, Concord Repatriation General Hospital, Sydney, New South Wales, Australia; Department of Gastroenterology, Tan Tock Seng Hospital, Singapore, Singapore; Department of Gastroenterology and Liver Services, Concord Repatriation General Hospital, Sydney, New South Wales, Australia; Faculty of Medicine and Health, University of Sydney, Sydney, New South Wales, Australia; Faculty of Medicine and Health Sciences, Macquarie University, Sydney, New South Wales, Australia; Faculty of Medicine and Health Sciences, Macquarie University, Sydney, New South Wales, Australia; Department of Gastroenterology and Hepatology, Blacktown and Mount Druitt Hospital, Sydney, New South Wales, Australia; Blacktown Clinical School, Western Sydney University, Sydney, New South Wales, Australia; Department of Gastroenterology and Liver Services, Concord Repatriation General Hospital, Sydney, New South Wales, Australia; Department of Gastroenterology and Liver Services, Concord Repatriation General Hospital, Sydney, New South Wales, Australia; Faculty of Medicine and Health, University of Sydney, Sydney, New South Wales, Australia; Faculty of Medicine and Health Sciences, Macquarie University, Sydney, New South Wales, Australia

**Keywords:** ulcerative colitis, vedolizumab, clinical remission, modeling

## Abstract

**Background and Aims:**

Vedolizumab is s gut-selective advanced therapy that is safe and efficacious for the treatment of ulcerative colitis (UC). Once patients achieve successful induction, there is a risk of loss of response leading to eventual flare. We aimed to identify these predictive factors and develop a practical scoring system to determine the ongoing efficacy of vedolizumab.

**Methods:**

We performed logistic regression on prospectively recruited UC subjects from the Vedolizumab Immunomodulator Enforced Withdrawal Study (VIEWS). All patients were in corticosteroid-free clinical remission and endoscopic improvement at baseline and continued vedolizumab. Predictive factors of 2-year corticosteroid-free clinical remission were determined and modeled into the VIEWS score, then validated in a real-world UC cohort.

**Results:**

Of 62 patients in the derivation cohort, 48 (77.4%) maintained clinical remission over two years. The predictive factors of remission were female (odds ratio [OR] 6.0, 95% confidence interval [CI], 1.2-29.7), antitumor necrosis factor naive (OR 3.8, 95% CI,1.0-14.0), baseline histological remission (OR 10.8, 95% CI, 2.4-48.4), thiopurine combination (OR 3.6, 95% CI, 0.7-18.0), and fecal calprotectin level ≤250 µg/g (OR 6.3, 95% CI, 0.9-42.2). These factors were incorporated into VIEWS score, yielding an area under the receiver-operating characteristic (AUROC) curve of 0.89 (95% CI, 0.81-0.98) in the prediction of 2-year clinical remission. Of 64 UC patients in the validation cohort, 40 (62.5%) remained in clinical remission at 2 years with AUROC of 0.77 (95% CI, 0.60-0.94). At the cut-off threshold of 4, the VIEWS score identified 2-year clinical remission with a sensitivity of 88.4% and specificity of 63.6%.

**Conclusions:**

Our study determined predictive factors and proposed a scoring system of ongoing clinical remission in UC patients on maintenance vedolizumab. In patients at high risk of relapse, combination therapy with thiopurine may be beneficial.

## Graphical Abstract

**Graphical Abstract:**
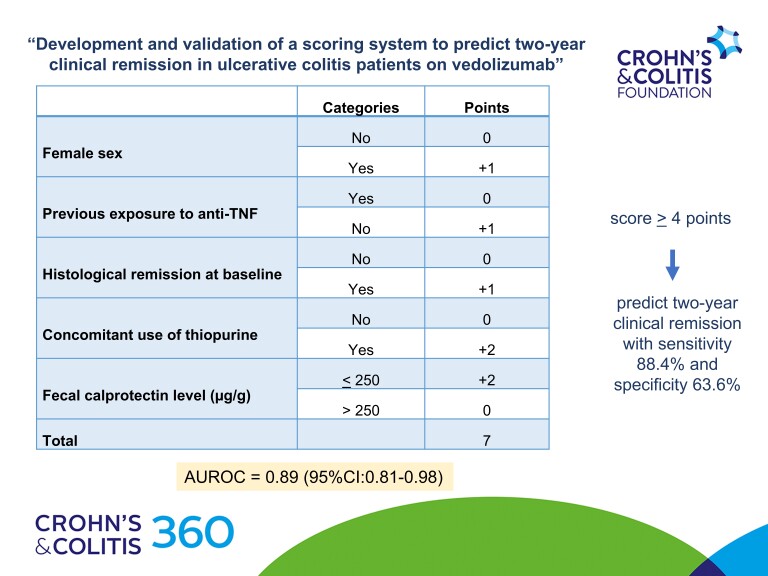

## Introduction

Ulcerative colitis (UC) is an intestinal disease characterized by chronic inflammation of the colon that results in substantial morbidity and disability.^1,2^ Patients with moderate-to-severe disease commonly require induction and maintenance treatment with an advanced therapy, which helps reduce inflammatory burden and normalize quality of life.^3^ Additionally, the absence of a definitive cure typically necessitates long-term treatment.^4^

Vedolizumab, a monoclonal antibody that selectively targets the α4β7 dimer and blocks the gut lymphocyte trafficking,^5^ has 47.1% clinical response and 41.8% clinical remission rate at week 52 in the placebo-controlled GEMINI 1 study^6^ with ongoing efficacy to 3 years.^7^ Recent meta-analysis also showed comparable estimate rates of clinical remission as 40% and 45% during induction and maintenance.^8^ However, one-third of patients do not enter primary response after induction, and a loss of response of 40 per 100 person-years of follow-up has been reported in those who primarily entered remission.^9^ Vedolizumab remains the foremost anti-integrin approved for the treatment of inflammatory bowel disease (IBD), esteemed for its gut selectivity, near absence of systemic adverse effects,^10^ and high persistence.^11,12^ Identifying factors associated with high response rates and remission rates may assist prescribers in the selection of advanced therapies.^13^ One scoring system derived from the GEMINI 1 dataset, the Clinical Decision Support Tool (CDST), has been shown to predict corticosteroid-free clinical remission (CSFCR) after 52 weeks of vedolizumab treatment.^14^ The CDST was developed during the era focusing on clinical endpoints; however, there need to be newer scoring systems based on the objective endpoints.^15^ Additionally, vedolizumab therapeutic drug monitoring and dose escalation are not effective in the prospective controlled ENTERPRET trial.^16^ Therefore, the identification of factors associated with flares might allow for optimization strategies. Conversely, factors associated with long-term clinical remission on vedolizumab might indicate good long-term prognosis, allow for de-escalation of treatment, and provide reassurance to patients and prescribers of not needing to switch out-of-class to a systemically active advanced therapy.

The aims of this study were to determine the predictive factors associated with CSFCR during vedolizumab maintenance therapy through a prospective longitudinal cohort study, develop a scoring system that can be easily used, and validate these factors against a second independent UC cohort. We anticipated that this study may provide clinicians with a valuable strategy to determine the prognosis in the use of vedolizumab for UC treatment.

## Materials and Methods

### Patients

From 2018 to 2022, a multicenter randomized controlled trial, Vedolizumab Immunomodulator Enforced Withdrawal Study (VIEWS),^17^ was conducted in Australia. Patients with UC ≥18 years treated with combination therapy of vedolizumab and thiopurine were recruited. Eligible participants were required to be in corticosteroid-free clinical remission (partial Mayo score ≤2 with no subscore >1) for at least 6 months and in endoscopic remission/improvement with a Mayo endoscopic score of 0-1. Patients with a history of corticosteroid use in the last 6 months, those currently on dual biologic therapy, Janus kinase inhibitor, or clinical trial drugs were excluded. After informed consent, participants were randomized at a ratio of 2:1 to either withdrawal or continuation of their thiopurine while on vedolizumab.

### Endpoints

Recruited subjects were monitored clinically for 2 years. Maintenance of CSFCR at 2 years of treatment was assessed. Predictive factors associated with 2-year CSFCR were identified comparing subjects that remained in remission versus those that developed clinical relapse during the study period. Sustained CSFCR over a 2-year period was defined as a partial Mayo score of ≤2 points, with no subscore >1 point, and being off corticosteroids. In those who failed to maintain CSFCR, additional confirmation of relapse was performed by fecal calprotectin and colonoscopy.

### Statistical Analyses and Ethical Approval

Parametric continuous variables were described as mean ± standard deviation and compared using *t*-test. Nonparametric continuous variables were described as median and interquartile range, and compared using the Mann–Whitney *U* test. Categorical data were analyzed using the chi-square or Fisher’s exact test, where appropriate. Univariable logistic regression was computed for each of the preselected factors. Continuous variables were transformed into binary categorization. A multiple logistic regression model was then constructed by including clinically meaningful factors associated with 2-year clinical remission in the univariate analysis, expressed as odds ratios (ORs) and their 95% confidence intervals (CIs) with a *P-*value cut-off of 0.1. A simplified scoring system (termed VIEWS Score) was developed converting regression coefficient into integer points and assigning these points to each level of each predictor. Each item score was summed to generate a linear composite score to predict the likelihood of 2-year clinical remission. Calibration plot and goodness-of-fit test were performed to assess the agreement and fitness between prediction and observation. The capability of the score to correctly define clinical remission was evaluated by using the area under the receiver-operating characteristic (AUROC) curve. The optimal cut-off was selected to maximize the diagnostic performance. The CDST scores of each patient in the validation cohort were also calculated and analyzed for diagnostic performance. All analyses were performed using STATA 17.0 (Stata Corporation).

All patients provided written informed consent. The study was approved by the Sydney Local Health District Human Research Ethics Committee (HREC/17/CRGH/22) and registered with the Australian and New Zealand Clinical Trials Registry (ACTRN12618000812291).

### External Validation Cohort

We intentionally recruited consecutive subjects on vedolizumab maintenance therapy. Patients in the validation cohort were from the real world, receiving vedolizumab reimbursed by the Australian Pharmaceutical Benefits Scheme. These real-world patients are permitted to remain on vedolizumab indefinitely should they remain in clinical remission, which requires gastroenterologist consultation every 3-6 months. These subjects underwent regular testing for biomarkers of inflammation as well as colonoscopy with assessments. UC patients aged ≥18 years exposed to vedolizumab from Macquarie University Hospital, Strathfield Private Hospital, Sydney Adventist Hospital, and Concord Hospital, Sydney, Australia, were recruited in this independent validation cohort if taking vedolizumab as maintenance therapy and had a follow-up period of ≥2 years.

## Results

### Patient Characteristics

The derivation cohort comprised 62 UC subjects (42% females, mean age 43.4 years). The validation cohort comprised 4 independent UC subjects (47% females, mean age 43.3 years) treated with vedolizumab (Table 1). Compared to the derivation cohort, the validation cohort had similar disease duration, prior exposure to corticosteroids, and antitumor necrosis factors (anti-TNF). Although participants in the validation cohort were selected from those who were on maintenance treatment, they had a higher inflammatory burden compared to the derivation cohort because of consecutive recruitment from real-world practice, whereas the VIEWS study excluded subjects not in deep remission. The validation group had a significantly higher proportion of subjects on corticosteroids, an exclusion factor for the VIEWS study. However, the rate of 2-year clinical remission was comparable between the 2 groups.

**Table 1.** Comparison between the derivation cohort and validation cohort.

	Derivation cohort (*N* = 62)	Validation cohort (*N* = 64)	*P-*value
Age—year	43.4 ± 18.0	43.3 ± 18.5	.97
Female sex	26 (41.9)	30 (46.9)	.58
Current or ex-smoker	16 (25.8)	10 (15.6)	.18
Disease extent			
Left-sided colitis	41 (66.1)	42 (65.6)	.95
Pancolitis	21 (33.9)	22 (34.4)	
Median UC duration—year	7.5 (4-12)	5 (3-9)	.06
Prior corticosteroid use	57 (91.9)	55 (85.9)	.39
Prior immunomodulator	62 (100)	58 (90.6)	.03
Prior anti-TNF	14 (22.6)	22 (34.4)	.17
Endoscopic improvement/remission^a^	54 (100)	40 (62.5)	<.001
Histological remission^b^	41 (75.9)	24 (38.1)	<.001
Baseline hemoglobin (g/L)	138.5 ± 13.7	131.9 ± 14.4	.02
Baseline fecal calprotectin (μg/g)	23.7 (7.9-65)	62 (17.6-193)	<.001
Baseline serum albumin (g/L)	41.4 ± 4.5	40.5 ± 6.0	.35
Baseline C-reactive protein (mg/L)	1.0 (0-2.5)	2.7 (0.8-6.6)	<.001
Concomitant thiopurine use	20 (32.3)	33 (51.6)	.03
Concomitant corticosteroid use	0 (0)	27 (42.2)	<.001
Rate of 2-year clinical remission	48/62 (77.4)	40/64 (62.5)	.08
Rate of 2-year clinical remission in thiopurine combination group	18/20 (90.0)	24/33 (72.7)	.18
Rate of 2-year clinical remission in vedolizumab monotherapy group	30/42 (71.4)	16/31 (51.6)	.08

Values are *N* (%), mean ± SD, or median (interquartile range).

Abbreviations: anti-TNF, antitumor necrosis factor; SD, standard deviation; UC, ulcerative colitis.

^a^ *Endoscopic improvement/remission* at baseline was defined as Mayo endoscopic score of 0-1. The percentage described is based on the total number of patients who had a screening endoscopy.

^b^ *Histological remission* at baseline was defined as Nancy histological index 0 (no or mild chronic inflammatory infiltrate).

### Predictive Factors and Development of a Simplified Scoring System

Out of 62 subjects, 48 (77.4%) remained in clinical remission after a 2-year follow-up. On univariable analysis, the following predictors were found to be significantly associated with achieving 2-year CSFCR on vedolizumab treatment (*P* ≤ .1): female sex (OR 6.0, 95% CI, 1.2-29.7), no previous anti-TNF exposure (OR 3.8, 95% CI, 1.01-14.0), histological remission defined by Nancy histological index 0 (OR 10.8, 95% CI, 2.4-48.4), concomitant use of thiopurine (OR 3.6, 95% CI, 0.7-18.0), and fecal calprotectin level ≤250 µg/g (OR 6.3, 95% CI, 0.9-42.2). Subsequent multivariable analysis was performed using clinical and biochemical variables from the univariable analysis (Table 2). A backward elimination approach was attempted, anti-TNF naïve status and female sex were not statistically significant while other significant factors in univariable analysis remained in the model. Since the parameters in univariable analysis were clinically meaningful, they were added to the model. We also analyzed the data including and excluding sex factor from the model as this is a novel predictive factor and had not been stated in previous literature. Data in Table 2 show the analysis including female sex. The model excluding female factor can be found in Supplementary Material. The final logistic regression model is as follows: log odds of 2-year clinical remission = −5.5152 + (1.8746 if sex was female) + (1.5954 if no previous anti-TNF exposure) + (1.8431 if baseline histology was in remission) + (3.2483 if thiopurine is concomitantly continued) + (3.6094 if fecal calprotectin ≤250 µg/g). Based on the final regression model, we developed a simple score by dividing regression coefficients with the smallest coefficient and rounded into integer points to serve as item scores. The total score ranged between 0 and 7, which was calculated by summing all the 5 predictive factors shown in Table 3.

**Table 2.** Univariable and multivariable analyses for corticosteroid-free clinical remission of the derivation cohort after 2-year maintenance treatment with vedolizumab.

	Remission at 2 years (*N* = 48)	Relapse at 2 years (*N* = 14)	Univariable analysis			Multivariable analysis	
			OR (95% CI)	*P-*value	AUROC (95% CI)	OR (95% CI)	*P-*value
Age—year	43.7 ± 16.8	42.4 ± 22.4	1.00 (0.97-1.0)	.80	0.54 (0.34-0.74)		
Female sex	24 (50)	2 (14.3)	6.00 (1.21-29.73)	.03	0.68 (0.56-0.80)	6.52 (0.77-55.12)	.08
Current/ex-smoker	13 (27.1)	3 (23.1)	1.24 (0.29-5.22)	.77	0.52 (0.38-0.66)		
Disease extent							
Left-sided colitis	32 (66.7)	9 (64.3)	0.90 (0.26-3.13)	.87	0.51 (0.34-0.63)		
Pancolitis	16 (33.3)	5 (35.7)					
UC duration—year	6.5 (3-10)	9 (6-17)	0.95 (0.89-1.02)	.16	0.66 (0.51-0.81)		
Prior corticosteroid	44 (91.7)	13 (92.9)	0.85 (0.09-8.25)	.89	0.49 (0.41-0.57)		
No previous anti-TNF exposure	40 (83.3)	8 (57.1)	3.75 (1.01-13.95)	.04	0.63 (0.49-0.78)	4.93 (0.57-42.86)	.15
Histological remission at baseline	37 (86.1)	4 (36.4)	10.79 (2.41-48.42)	<.01	0.75 (0.59-0.91)	6.32 (0.98-40.90)	.05
Concomitant thiopurine	18 (37.5)	2 (14.3)	3.60 (0.72-17.96)	.10	0.62 (0.50-0.73)	25.75 (1.26-526.62)	.04
Serum CRP ≤5 mg/L	42 (87.5)	12 (85.7)	1.17 (0.21-6.54)	0.86	0.51 (0.40-0.62)		
Fecal calprotectin ≤250 µg/g	46 (95.8)	11 (78.5)	6.27 (0.93-42.20)	0.05	0.59 (0.47-0.70)	36.94 (1.22-1117.02)	.04

Values are *N* (%), mean ± SD, or median (interquartile range).

Abbreviations: anti-TNF, antitumor necrosis factor; AUROC, area under the receiver-operating characteristic curve; CI, confident interval; CRP; C-reactive protein; OR, odds ratio; UC, ulcerative colitis.

**Table 3.** Development of simplified scoring system based on final regression model.

Predictors	Categories	Reference value	Regression coefficients	Points
Female sex	No	0	1.8746	0
	Yes	1		1
Previous exposure to anti-TNF	Yes	0	1.5954	0
	No	1		1
Baseline histological remission	No	0	1.8431	0
	Yes	1		1
Concomitant use of thiopurine	No	0	3.2483	0
	Yes	1		2
Fecal calprotectin level	≤250 µg/g	1	3.6094	2
	>250 µg/g	0		0
Total score				7

Abbreviation: anti-TNF, antitumor necrosis factor.

### Performance of the New Simplified Predictive Scoring System and Optimal Cut-Off Selection

**Figure 1.**
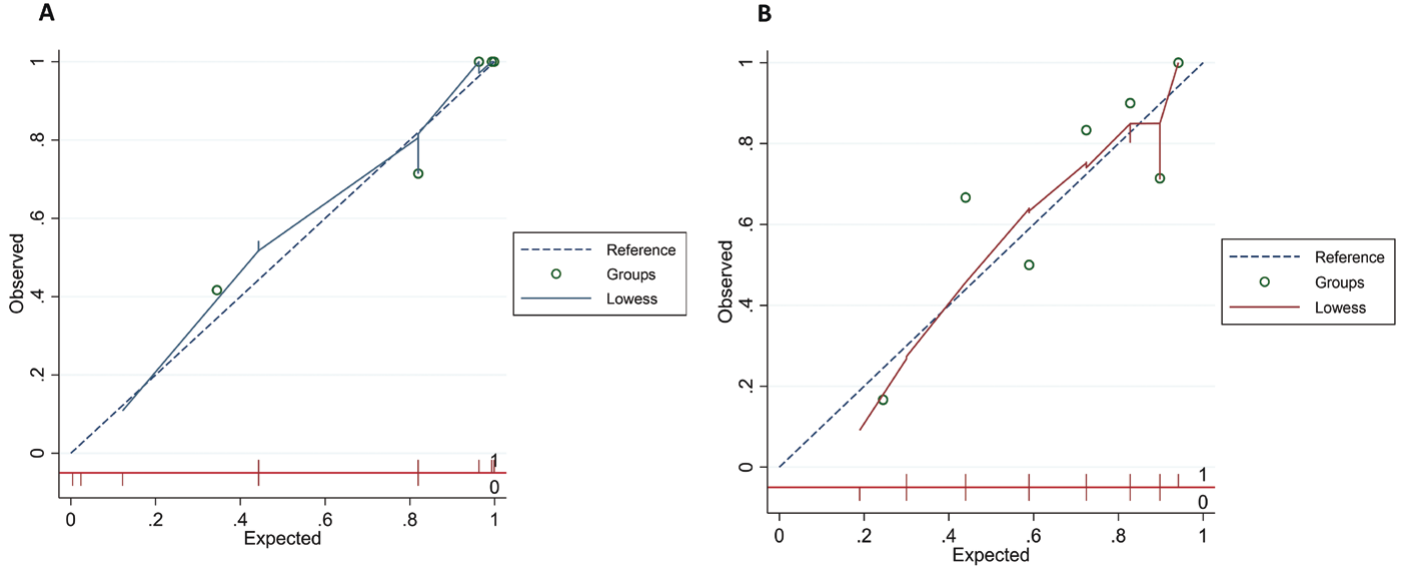
Calibration plot for derivation (A) and validation (B) cohort.

**Figure 2.**
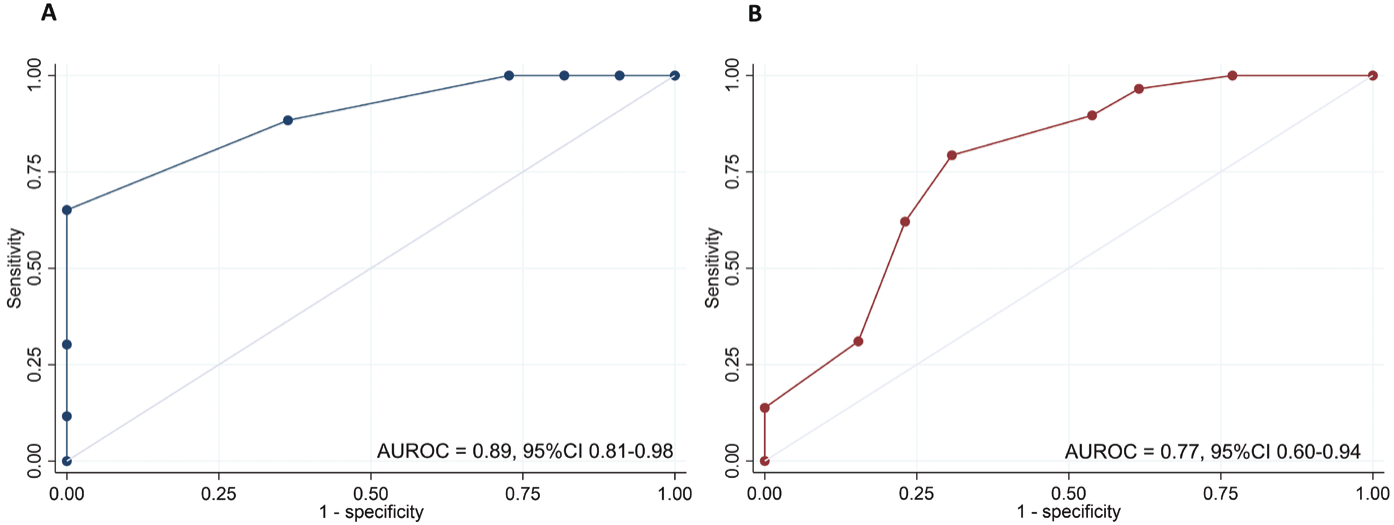
The area of receiver-operating characteristic curve of the models in derivation (A) and validation (B) cohort.

The internal calibration plot showed close alignment with the 45-degree line, indicating strong agreement with the derivation model (Figure 1A), and no evidence of poorly fit data (Pearson goodness-of-fit test *P-*value .89). The excellent discrimination ability of the model in determining 2-year CSFCR was evident (AUROC 0.89, 95% CI, 0.81-0.98; Figure 2A). The optimal cut-off value of 4 was determined with a sensitivity of 88.4%, specificity of 63.6%, positive predictive value (PPV) of 90.5%, negative predictive value (NPV) of 58.3%, positive likelihood ratio (LR+) of 2.43, and negative likelihood ratio (LR−) of 0.18. The diagnostic performance of the score is presented in Table S1.

### Performance of the New Predictive Scoring System and CDST After External Validation

After applying to the validation cohort, the model calibration plot falls along a diagonal line (Figure 1B), with AUROC of 0.77 (95% CI, 0.60-0.94; Figure 2B). At the same cut-off, the sensitivity, specificity, PPV, NPV, LR+, and LR− were 79.3%, 69.2%, 85.2%, 60.0%, 2.58, and 0.30, respectively.

The CDST that incorporates disease duration, prior anti-TNF, baseline moderate endoscopic activity, and baseline albumin concentration, with a cut-off of 32 indicating a higher probability of response to vedolizumab was applied to our validation cohort. Using this cut-off point, the sensitivity and specificity were 54.5% and 59.1%, respectively (Table 4).

**Table 4.** Diagnostic performances of the CDST and the VIEWS Score in determining maintenance of remission in validation cohort patients with ulcerative colitis on vedolizumab.

Predicting tools for corticosteroid-free clinical remission	Sensitivity (95% CI) (%)	Specificity (95% CI) (%)	Positive likelihood ratio (95% CI)	Negative likelihood ratio (95% CI)
CDST score^a^ ≥ 32	54.5 (36.4-71.9)	59.1 (36.4-79.3)	1.33 (0.74-2.41)	0.77 (0.46-1.28)
VIEWS score ≥ 4	79.3 (60.3-92.0)	69.2 (38.6-90.9)	2.58 (1.12-5.95)	0.30 (0.13-0.66)
VIEWS score (excluding female sex) ≥ 4	72.4 (52.8-87.3)	76.9 (46.2-95.0)	3.14 (1.13-8.68)	0.36 (0.19-0.69)

Abbreviations: anti-TNF, antitumor necrosis factor; CDST, Clinical Decision Support Tool; CI, confident interval; UC, ulcerative colitis; VIEWS, Vedolizumab Immunomodulator Enforced Withdrawal Study.

^a^ *CDST to predict outcomes of vedolizumab therapy in UC* is summation of +3 points if disease duration ≥2 years, +3 points if no prior anti-TNF, +2 points if baseline endoscopy moderate activity, and +0.65 points per 1 g/L of baseline albumin concentration.

## Discussion

In this study, we analyzed prospectively recruited UC patients treated with vedolizumab who had corticosteroid-free deep remission at baseline. Next, we identified independent factors that were associated with ongoing remission based on protocolized clinical endpoints over 2 years. We then developed a simple scoring system that represented the likelihood of remaining in remission during long-term vedolizumab treatment. The model was validated against a second independent cohort and retained good discrimination ability. At a cut-off level of 4, this tool demonstrated 88.4% sensitivity and 63.6% specificity in identifying UC patients likely to remain in CSFCR.

The 5 predictive factors of ongoing remission in UC are (1) bio-naïve status to anti-TNF; (2) female sex; (3) concurrent thiopurine use; (4) Nancy Histological Index = 0; and (5) fecal calprotectin ≤250 µg/g. Of these factors, the controllable factor is the continuation of thiopurine. In theory, thiopurine co-therapy may help reduce antidrug antibody formation thereby increasing the serum biologic trough concentrations, as demonstrated in subjects on anti-TNF therapy.^18^ However, for vedolizumab, the VIEWS study showed no difference in the serum trough levels between those who continued or ceased thiopurine.^17^ Therefore, serum vedolizumab trough levels may not be a factor in the prediction of maintenance of remission. While vedolizumab is less immunogenic, combining this with a thiopurine may offer non-pharmacokinetic advantages, including added immunosuppressive effects. Although thiopurines are rarely associated with the development of lymphoma, skin cancer, hepatotoxicity, and myelotoxicity, these risks may need to be counterbalanced against loss of response to vedolizumab. Therefore, patients with lower risk factors may be safely de-escalated to vedolizumab monotherapy. Alternatively, subjects with poorer prognostic scores may benefit from continuation of thiopurine, or until they have entered histological remission which was demonstrated in the model to reduce the risk of subsequent flare. Subjects with favorable features of females, without prior anti-TNF exposure, in histological remission, and with low fecal calprotectin levels, are most likely to be able to cease thiopurine and continue vedolizumab monotherapy. Previously, in the absence of evidence, the continuation or cessation of thiopurine in vedolizumab subjects was largely haphazard. Unlike anti-TNF failure for which intraclass switching is possible, vedolizumab failure is consequential. Vedolizumab remains the only anti-integrin approved for use in IBD and remains the preferred drug of many prescribers especially for IBD patients with comorbidities, older age, prior malignancies, and prior sepsis.^19^ This is the first study to objectively determine factors that can prognosticate UC patients on vedolizumab. For example, consider a 50-year-old female who is in stable clinical remission on vedolizumab as the first biologic therapy and thiopurine, with fecal calprotectin of 50 µg/g, resulting in a VIEWS score of 6. This high score allows clinicians to reassure this patient about ongoing remission and confidently discuss the possibility of thiopurine discontinuation. In this scenario, if the patient is concerned about the long-term risk of thiopurine, this score can ensure clinicians in identifying good candidates for safe de-escalation, while continuing vedolizumab monotherapy with close monitoring.

This study evaluated a cohort of UC subjects in remission to identify which subjects relapsed and required treatment escalation. Our study, therefore, contrasts with prior studies that aimed to determine the predictors of inducing treatment response with vedolizumab. The CDST was validated on subjects with active UC with Mayo score ≥6, which helped predict successful induction of remission.^6,14^ In testing for subjects in the maintenance of remission, however, it was not as sensitive or specific as the VIEWS Score. The CDST shared anti-TNF exposure status with the VIEWS score to be associated with treatment efficacy. Anti-TNF bio-naïve status tends to have a significantly better long-term response to vedolizumab than those who are bio-exposed to anti-TNF.^14,20^ Furthermore, the VIEWS score identified other components not shared with the CDST. Female sex was a novel factor identified in this study that was also confirmed in the independent validation cohort. Our modeling showed that the scoring system remained valid both including and excluding the sex factor, which yielded similar AUROC in both the derivation and validation cohorts. It remains uncertain if this is due to a biological effect or from confounding factors such as differences in drug clearance.

The fourth factor, Nancy histological index 0, served as a significant predictive factor for clinical remission. This is one of the first studies to prospectively demonstrate the importance of histological remission combined with deep remission or “disease clearance” in UC. Prior to this, the benefits of histological remission had been primarily derived from retrospective cohort studies.^21^ This is the first prospective interventional study that included histological status at baseline to determine the outcomes after two years. Previous studies have focused on endoscopic activity in the prediction of outcomes^14,22^ but not histological activity in the setting of endoscopic remission. The last factor, fecal calprotectin ≤250 µg/g, was incorporated into the model due to its noninvasive nature, serving as a proxy for recent mucosal improvement. Previous prospective studies demonstrated its ability to predict long-term outcomes following vedolizumab treatment.^23^

Key strengths of this study included the prospective cohort design that established data collection over 2 years. All subjects were followed up and treatment escalation was well defined and verified using objective indicators. Adherence to thiopurines was checked using therapeutic drug monitoring. Multiple IBD centers recruited subjects that increased generalizability. The scoring system was validated against a second independent cohort, which was a real-world cohort of vedolizumab patients, and it supported the role of the VIEWS score in identifying those likely to remain in clinical remission. Another strength of the study is as one of the first prospective controlled studies to demonstrate the importance of histological remission and disease clearance in improving clinical outcomes.

There are some limitations of the study. The sample size of the VIEWS study, calculated based on the difference of target vedolizumab trough concentrations between 2 groups, was relatively small which may affect the generalizability. However, the derivative data demonstrated sufficient statistical power to identify better versus poorer patient outcomes based on objective assessments, all evaluated by a blinded assessor. The findings achieved from this cohort were validated against an independent cohort and identified similar performance. Second, this scoring system was developed based on specific individuals who were on combination therapy before enrollment. However, we then tested the scoring system in a real-world validation cohort in which some patients were on vedolizumab monotherapy. Lastly, although all patients in the validation cohort achieved clinical remission, not all of them attained endoscopic remission. Further validation on additional cohorts may be needed to further confirm the external validity.

In conclusion, we identified favorable predictive factors, developed, and validated a practical predictive scoring system to determine UC patients with a high likelihood of sustained clinical remission following vedolizumab treatment. These factors appear to be independent from those that are useful for the predictive efficacy of induction therapy. The VIEWS score found combination thiopurine therapy with vedolizumab to improve ongoing clinical remission, which might be relevant for some patients with higher risk profiles for disease relapse. Conversely, histological remission is strongly associated with sustained remission, so de-escalation of combination therapy may be recommended.

## Supplementary Material

otae068_suppl_Supplementary_Table_S1-S2_Figure_S1-S2

## Data Availability

Data are provided in the Supplementary Material and also available to researchers upon request.
