# Supplementary material for: Development and Validation of a Scoring System to Predict 2-Year Clinical Remission in Ulcerative Colitis Patients on Vedolizumab
Source: Crohns Colitis 360. 2024 Dec 28;7(1):otae068. doi: 10.1093/crocol/otae068 (PMC11700619; doi:10.1093/crocol/otae068)
Supplement: otae068_suppl_Supplementary_Table_S1-S2_Figure_S1-S2 [file otae068_suppl_supplementary_table_s1-s2_figure_s1-s2.docx]

**Supplementary material**

**Table S1:** Diagnostic performance of each cut-off point of the proposed scoring system

| **Cut-off point** | **Sensitivity** (%) | **Specificity** (%) | **Positive likelihood ratio** | **Negative likelihood ratio** |
| --- | --- | --- | --- | --- |
| 0 | 100 | 0 | 1.0 | - |
| 1 | 100 | 9.09 | 1.10 | 0 |
| 2 | 100 | 18.2 | 1.22 | 0 |
| 3 | 100 | 27.3 | 1.38 | 0 |
| 4 | 88.4 | 63.6 | 2.43 | 0.18 |
| 5 | 65.1 | 100 | - | 0.35 |
| 6 | 30.2 | 100 | - | 0.70 |
| 7 | 11.6 | 100 | - | 0.88 |

**A model predicting two-year corticosteroid-free clinical remission on vedolizumab maintenance treatment (excluding sex factor)**

We analyzed the model excluding sex factor and final logistic regression model was as follows: log odds of two-year clinical remission = -4.4553 + (1.5954 if no previous anti-TNF exposure) + (2.0575 if baseline histology was in remission) + (2.3876 if thiopurine is concomitantly continued) + (3.2004 if fecal calprotectin <250 µg/g). Utilizing four factors, the total score ranged between 0 to 6 points which was calculated by adding previous exposure to anti-TNF, yes (0 point) or no (1 point), baseline histological remission, no (0 point) or yes (1 point), concomitant use of thiopurine, no (0 point) or yes (2 points), and fecal calprotectin level >250 µg/kg (0 point) or < 250 µg/g (2 points). The internal calibration plot **(Figure S1A)**, Pearson goodness-of-fit test showed good agreement, and excellent discrimination with AUROC of 0.85 (95% CI 0.73-0.97) **(Figure S2A).** Choosing cut-off point of 4, the sensitivity, specificity, positive predictive value, negative predictive value, positive likelihood ratio, and negative likelihood ratio of the model was 83.7%, 72.7%, 92.3%, 53.3%, 3.07, and 0.22, respectively. The sensitivity, specificity, positive, and negative likelihood ratio by each cut-off points are presented in **Table S2**. Then, we applied the model into the validation cohort, the calibration plot showed acceptable agreement, shown in **Figure S1B,** and the AUROC was 0.82 (95%CI 0.67-0.98) **(Figure S2B)**. At the same cut-off, the sensitivity, specificity, positive predictive value, negative predictive value, positive likelihood ratio, and negative likelihood ratio were 72.4%, 76.9%, 87.5%, 55.6%, 3.14, and 0.36, respectively.

**Figure S1:** Calibration plot for derivation (S1A) and validation (S2B) cohort excluding sex factor

**A**

**B**

**Table:** Sensitivity and specificity of scoring system excluding female sex

**Figure S2:** The area of receiver operating characteristic curve of the model in derivation (S2A) and validation (S2B) cohort excluding sex factor

**A**

**B**

**Table S2:** Diagnostic performance of each cut-off point of the scoring system excluding sex factor

| **Cut-off point** | **Sensitivity** (%) | **Specificity** (%) | **Positive likelihood ratio** | **Negative likelihood ratio** |
| --- | --- | --- | --- | --- |
| 0 | 100 | 0 | 1 | - |
| 1 | 100 | 9.09 | 1.10 | 0 |
| 2 | 100 | 18.2 | 1.22 | 0 |
| 3 | 97.7 | 36.4 | 1.54 | 0.06 |
| 4 | 83.7 | 72.7 | 3.07 | 0.22 |
| 5 | 32.6 | 100 | - | 0.67 |
| 6 | 30.2 | 100 | - | 0.70 |
